# The Capacity Crunch: Auditing Mental Capacity Assessments in the Emergency Department

**DOI:** 10.1192/bjo.2025.10180

**Published:** 2025-06-20

**Authors:** Maria Sarang, Freya Carter

**Affiliations:** University Hospitals Southampton, Southampton, United Kingdom

## Abstract

**Aims:** To evaluate the level of documentation and quality of mental capacity assessments (MCA) in the Emergency Department (ED), specifically examining the frequency, documentation methods, and outcomes of capacity assessments for patients presenting with mental health complaints. We hypothesize that most patients who attend with mental health presentations and leave before treatment is completed do not receive formal capacity assessments.

**Methods:** A retrospective audit was conducted of all patients attending ED triaged under the “Mental Health” category during October 2024 (n=81). Data was collected on demographics, presenting complaints, rates of re-attendance, whether formal and informal capacity assessments were carried out, involvement of Liaison Psychiatry and patient outcomes. Formal capacity assessments were defined as those using the MCA form or explicitly documenting the decision in question, reason for doubt, and assessment of the four key criteria (understanding, retaining, weighing, and communicating information).

**Results:** Of 81 patients (49% female, median age 29, range 13–77), the predominant presenting complaints were suicidal ideation (n=33, 41%), overdose (n=9, 11%), and depressed mood (n=8, 10%). 75 patients (92.6%) left before treatment was completed. 16 (21.3%) of those who left before treatment was completed returned within 24 hours.

Only 7.4% (n=6) had formal capacity assessments documented, with 42% (n=34) having informal assessments noted elsewhere. 5 of 6 formal assessments were done by ED staff and one was conducted by Liaison Psychiatry staff. Of all assessments conducted (n=40), 8 patients (20%) lacked of capacity at the time. The majority of patients (92.6%) left before treatment completion. Liaison Psychiatry was involved in 34.6% (n=28) of cases.

**Conclusion:** This audit highlights significant gaps in the formal documentation of capacity assessments in the ED, with few mental health presentations receiving fully documented assessments despite RCEM and MCA guidance. The high rate of patients leaving before treatment completion underscores the need for further investigation into possible reasons, a standardized assessment approach to capacity assessment and focused training for ED staff. Informal assessments may be more common due to time pressures, limited knowledge of the MCA process, or difficulty accessing forms.

